# Conventional microdiscectomy versus unilateral biportal endoscopy for lumbar disc herniation during the learning curve: Propensity-score matched analysis of clinical results

**DOI:** 10.1016/j.bas.2025.105872

**Published:** 2025-11-19

**Authors:** Nicolas Ross, Matthieu Vassal, Alexandre Dhenin, Guillaume Lonjon

**Affiliations:** a Orthosud Montpellier; Clinique Saint Jean Sud de France, Montpellier, France; b Hospital Universitario Austral, Buenos Aires, Argentina

**Keywords:** Lumbar disc herniation, Microdiscectomy, Unilateral biportal endoscopy discectomy

## Abstract

**Introduction:**

Endoscopy approaches to lumbar disc herniation (LDH) surgery, particularly unilateral biportal endoscopy (UBE), have gained in popularity because of their minimally invasive nature and potential for good recovery. However, comparisons with conventional microdiscectomy, especially during learning curve, are limited.

**Research question:**

This study compared clinical outcomes, safety, and resource use between UBE and conventional microdiscectomy for LDH during the learning curve of UBE implementation.

**Material and methods:**

This retrospective, single-center study analyzed data for 363 patients who underwent LDH surgery from January 2022 to September 2023. After 1:1 propensity score matching, 302 patients (151 per group) were included. Patients were evaluated preoperatively and at 1, 3, and 12 months postoperatively with the Oswestry Disability Index (ODI), lumbar and radicular pain scales, and satisfaction surveys. Complications, reoperation rates, operative time, hospitalization, and disposable costs were also analyzed.

**Results:**

Both groups experienced significant improvement in ODI and pain scores at all time points, without significant differences between groups. Satisfaction and return-to-work rates were similarly high in both groups. Complication and reoperation rates did not differ significantly. The UBE group featured longer operative times (57 vs. 44 min) and higher disposable costs (€261 vs. €91) than conventional microdiscectomy but a higher outpatient discharge rate (20.5 % vs. 9.3 %).

**Discussion and conclusion:**

UBE is as effective and safe as conventional microdiscectomy for LDH, even during the learning curve. It allows for early adoption without compromising patient outcomes and may offer advantages in outpatient feasibility, despite longer operating times and higher procedural costs.

## Highlights

• ODI, pain scores and complications rates are similar after UBE or open LDH surgery.
• UBE had longer operative time and higher operating room costs.
• Higher ambulatory surgery rate may offset increased UBE operative costs.
• UBE is as safe and effective as conventional surgery for LDH, even in early cases.

## 1 Introduction

Surgical treatment of non-urgent lumbar disc herniation (LDH) is recommended if conservative measures fail after 6–12 weeks (Gibson and Waddell, 2007). It yields better clinical results than conservative treatment in the short and medium term (Kreiner et al., 2014). The original open discectomy technique evolved into microdiscectomy and has since been considered the standard technique for lumbar disc herniation (Alvi et al., 2018).

Over the last decade, we have witnessed an increase in the use of endoscopic techniques in spinal surgery. Supporters argue that these techniques provide better clinical results and shorter hospitalizations with enhanced visualization than the standard technique (Zheng et al., 2022). There are two main types of endoscopic techniques: monoportal endoscopy, with the camera and the channel for the instruments in the same instrument (a single 8-mm skin incision) and biportal endoscopy (unilateral biportal endoscopy [UBE]), with the camera and the channel for the instruments dissociated by a few centimeters (two 10-mm skin incisions). Both have advantages and disadvantages in the treatment of disc herniation. The advantages of UBE include a faster learning curve because of its greater similarity to conventional surgery and simpler adaptability than monoportal endoscopy (Sellier et al., 2024). However, monoportal techniques seem even less invasive than UBE because they require minimal workspace creation (Wu et al., 2023).

Although the use of endoscopy for treating LDH is increasingly being used in France and worldwide, few articles have been published on the subject, particularly for UBE (Chu et al., 2022; Lin et al., 2019). Recent meta-analyses comparing UBE surgery with microdiscectomy (Zheng et al., 2022; Lin et al., 2019) showed that clinical studies often have a short follow-up period (<1 year) (Jiang et al., 2022) or involve a small number of participants (<60 patients undergoing surgery with UBE for LDH) (Kim et al., 2018; Eun et al., 2017; Chang et al., 2023; Ahn et al., 2018). Furthermore, no studies have included cases involving the learning curve, reflecting real life transition from one technique to another.

This study compared clinical function in the medium and long term for patients undergoing LDH surgery with conventional microdiscectomy or UBE.

## 2 Methods

### 2.1 Study design

This was a single-center retrospective study conducted within a spinal surgery department in France, with data collected from January 2022 to September 2024.

The study received the approval of an institutional review board (accreditation no. 0990-0279; reference no. 20240103). The patients were informed about the use of their medical data for the study and were allowed to object to it.

### 2.2 Study population

All patients underwent surgery for LDH by one of three spine surgeons (GL, AD, MV) from January 2022 to September 2023. The UBE technique began to be used in the center in May 2022. During a few months, the three surgeons performed surgery with both techniques. The selection of the technique depended mainly on the type of herniation. At the beginning of the learning curve with UBE, simple cases were selected, mostly non-migrated left L5-S1 disc herniations. Progression to more complex cases occurred with increasing surgical volume. Data for all patients included in this learning curve period were analyzed. Non-inclusion criteria were LDH recurrence, prior surgery on the same level, and bilateral decompression in the same surgical act.

### 2.3 Surgery

All patients underwent LDH surgery under general anesthesia without the addition of local anesthetics. Surgery involved a conventional technique of open microdiscectomy with a mini retractor (control group, “Open”) or a UBE technique ("UBE" group). In the Open group, patients were placed in the knee–chest position to open the interlaminar window with use of a retractor and a microscope. The incision was 2 cm long, close to the midline on the side of the hernia. In the UBE group, patients were positioned in the prone position, and a 6-mm endoscope was used. Two 1-cm incisions were created approximately 3 cm apart, adhering to the midline on the side of the hernia.

Postoperative care did not differ between the two groups. Patients were discharged home the same day or the following day. They had no restrictions on daily activities, but they were not authorized to drive for 3 weeks or engage in sports for 8 weeks.

### 2.4 Clinical evaluation

Patients were clinically evaluated preoperatively and postoperatively at 1 month (M1), 3 months (M3), and 1 year (M12) by their surgeon, with an autoevaluation of their functional disability (Oswestry Disability Index [ODI]) and pain (visual analog scale [VAS]) for lumbar (L-VAS) and radicular (R-VAS) pain. Patient satisfaction was evaluated at M1 and M3.

A decrease of at least 12.8 points between postoperative and preoperative ODI values was considered a Minimal Clinically Important Difference (MCID) (Austevoll et al., 2019). All peri-operative complications and revision surgeries were collected during a minimum of 1 year.

### 2.5 Statistical analysis

To account for potential biases in baseline characteristics between groups, we used 1:1 propensity-score matching without replacement. Propensity scores were calculated by using a logistic regression model including the following covariates: age, sex, body mass index (BMI), American Society of Anesthesiologists (ASA) physical status score, preoperative ODI, and preoperative R-VAS.

Mean and frequency imputations were used in cases of missing data on matching covariates. Patients in the Open group were matched to patients in the UBE group by nearest-neighbor matching with a caliper value of 0.2 of the pooled standard deviation of the propensity score logs. Standardized mean differences (SMDs) were calculated to compare baseline characteristics after matching. A postmatching SMD <0.1 was considered an acceptable difference. The standard support assumption was assessed with the Kolmogorov-Smirnov non-parametric test. Common support intervals were determined with the trimming method and kernel density estimators. The threshold was set at 0.001. Statistical analysis involved using EasyMedStat 3.32.

Numerical variables are expressed as mean (±SD), and other variables as absolute and relative frequency (%). Normality and heteroscedasticity of continuous data were assessed with the Shapiro-Wilk and Levene tests, respectively. Continuous outcomes were compared with the unpaired Student's t-test, Welch’s *t*-test, or Mann-Whitney *U* test depending on the data distribution. Categorical outcomes were compared with the chi-squared test or Fisher's exact test. The alpha risk was set at 5 %, and two-tailed tests were used. P < 0.05 was considered statistically significant.

## 3 Results

### 3.1 Baseline characteristics before (Table 1) and after matching (Table 2)

In total 363 patients met the inclusion criteria (202 men; mean age 51.5 years): 183 underwent conventional surgery and 180 UBE surgery. Baseline patient characteristics differed in preoperative ODI and R-VAS, patients in the UBE group being significantly more disabled and painful than those in the Open group (Table 1).

**Table 1:** Demographic and preoperative data for the entire study population undergoing open microdiscectomy with a mini retractor (control group, “Open”) or a unilateral biportal endoscopy technique ("UBE") (n = 363).

	Open group n = 183	UBE group n = 180	p-value
Sex			
Males	104 (56.8 %)	98 (54.4 %)	0.725
Females	79 (43.2 %)	82 (45.6 %)	
Age (years)	52.0 (±14.3)	50.96 (±15.0)	0.705
BMI (kg/m^2^)	25.44 (±4.4)	26.56 (±5.0)	0.052
Obesity (BMI >30 kg/m^2^)	28 (17.6 %)	35 (13.8 %)	0.339
Sport practice	83 (50.3 %)	69 (43.4 %)	0.257
Tobacco use	64 (38.6 %)	58 (36.2 %)	0.889
Anticoagulant/antiplatelet use	11 (9.5 %)	12 (11.3 %)	0.819
Baseline L-VAS (/10)	5.9 (±2.6)	6.4 (±2.6)	0.07
Baseline R-VAS (/10)	7.0 (±2.1)	7.4 (±2.3)	0.043*
Baseline ODI (/100)	45.4 (±20.3)	49.9 (±18.9)	0.034*
ASA score			
1	97 (69. 8 %)	123 (68.3)	0.317
2	33 (23.7 %)	51 (28.3 %)	
3	9 (6.5 %)	6 (3.3 %)	
Hernia side			
Left	95 (51.9 %)	108 (60.0 %)	0.148
Right	88 (48.1 %)	72 (40.0 %)	
Hernia type			
Median/paramedian	163 (89.1 %)	166 (92.2 %)	0.395
Foraminal/extraforaminal	20 (10.9 %)	14 (7.8 %)	
Surgical approach			
Left/contralateral	95 (51.91 %)/1	111 (61.7 %)/3	0.077
Right/contralateral	88 (48.1 %)/1	69 (38.3 %)/0	
Operated level			
L1L2	2 (1.1 %)	1 (0.6 %)	0.082
L2L3	5 (2.7 %)	6 (3.3 %)	
L3L4	31 (16.9 %)	14 (7.8 %)	
L4L5	73 (39.9 %)	72 (40.0 %)	
L5S1	70 (38.3 %)	86 (47.8 %)	
L4S1	2 (1.1 %)	1 (0.6 %)	

BMI: body mass index; L-VAS: visual analog scale for lumbar pain; R-VAS: visual analog scale for radicular pain; ODI: Oswestry disability index; ASA: American society of anesthesiologists physical status score.

After using propensity scores and matching patients on age, sex, BMI, ASA score, preoperative ODI, and R-VAS, 302 patients were finally included in the analysis, 151 in each group (Table 2). The two groups did not differ in baseline patient characteristics. However, the left approach was predominantly used with the UBE versus Open technique (64.9 % vs. 53.2 %; p = 0.035).

**Table 2:** Demographic and preoperative data for study population after matching for the Open and UBE groups (n = 302).

	Open group n = 151	UBE group n = 151	SMD	p-value
Sex				
Masculine	83 (55.0 %)	81 (53.6 %)		
Feminine	68 (45.0 %)	70 (46.4 %)	−0.027	0.908
Age (years-old)	51.08 (±14.0)	51.17 (±15.2)	−0.0065	0.955
BMI (kg/m^2^)	25.79 (±4.5)	26.22 (±4.8)	0.023	0.578
Obesity (BMI >30 kg/m^2^)	31 (20.5 %)	28 (18.5 %)		0.772
Sport practice	70 (49.0 %)	63 (44.4 %)		0.511
Tobacco use	45 (31.3 %)	40 (28.0 %)		0.785
Anticoagulant/antiplatelet use	6 (6.0 %)	9 (9.6 %)		0.425
Baseline L-VAS (/10)	6.0 (±2.5)	6.2 (±2.6)		0.409
Baseline R-VAS (/10)	7.3 (±1.9)	7.2 (±2.3)	−0.033	0.646
Baseline ODI (/100)	47.5 (±19.7)	48.1 (±17.8)	−0.035	0.766
ASA score				
1	103 (68.2 %)	102 (67.6)		0.819
2	40 (26.5 %)	48 (28.5 %)		
3	8 (5.3 %)	6 (4.0 %)		
Hernia side				
Left	79 (52.3 %)	95 (62.9 %)		0.081
Right	72 (47.7 %)	56 (37.1 %)		
Hernia type				
Median/paramedian	132 (87.4 %)	139 (92.1 %)		0.255
Foraminal/extraforaminal	19 (12.6 %)	12 (8.0 %)		
Surgical approach				
Left/contralateral	79 (52.3 %)/1	98 (64.9 %)/3		0.035*
Right/contralateral	72 (47.7 %)/1	53 (35.1 %)/0		
Operated level				
L1L2	2 (1.3 %)	1 (0.7 %)		0.089
L2L3	3 (2.0 %)	6 (4.0 %)		
L3L4	24 (15.9 %)	10 (6.6 %)		
L4L5	61 (40.4 %)	60 (39.7 %)		
L5S1	60 (39.7 %)	73 (48.3 %)		
L4S1	1 (0.7 %)	1 (0.7 %)		

SMD: standardized mean differences; BMI: body mass index; L-VAS: visual analog scale for lumbar pain; R-VAS: visual analog scale for radicular pain; ODI: Oswestry disability index; ASA: American society of anesthesiologists physical status score.

### 3.2 Disability and pain

**Fig. 1:**
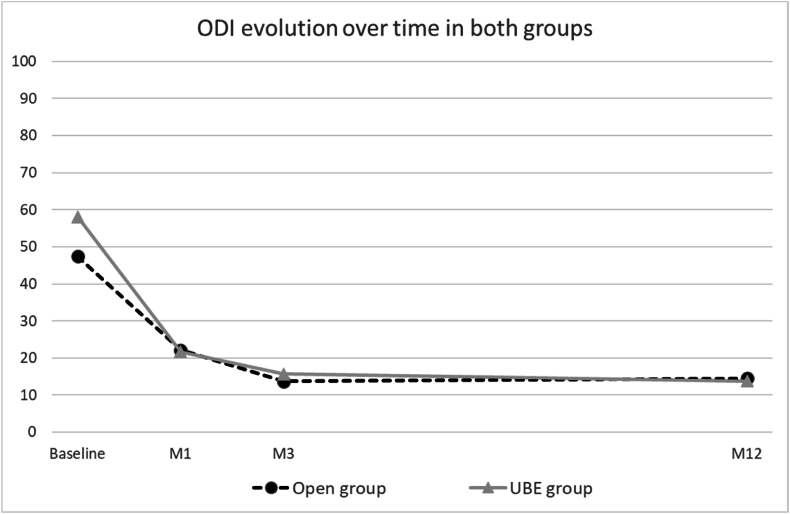
Mean evolution in Oswestry Disability Index (ODI) over time in the Open group and UBE group ODI: Oswestry disability index; M1: 1 month postoperatively; M3: 3 months postoperatively; M12: 1 year postoperatively.

For both groups, the ODI decreased significantly at M1 postoperatively. Clinical improvement continued from M1 to M3 postoperatively, followed by a plateau from M3 to M12 (Fig. 1), with no significant differences between the two groups at any time point.

Lumbar and radicular pain also decreased from M1 postsurgery to the end of follow-up. At M1, R-VAS was lower in the UBE than Open group (2.7 vs. 3.4/10) but not significantly (p = 0.052). This difference was no longer observed at M3 (R-VAS 2.5 vs. 2.6, p = 0.483) (Table 3, Table 4, Table 5).

**Table 3:** Clinical evaluation at 1 month postoperatively.

	Open group n = 151	UBE group n = 151	p-value
ODI (/100)	22.2 (±17.5)	21.7 (±15.9)	0.975
Mean ODI decrease	26.1 (±23.3)	26.5 (±20.6)	0.514
MCID reached	100 (71.9 %)	111 (81.0 %)	0.102
L-VAS (/10)	2.8 (±2.5)	2.9 (±2.2)	0.351
Mean L-VAS decrease	3.2 (±2.9)	3.3 (±2.9)	0.737
R-VAS (/10)	3.4 (±2.8)	2.73 (±2.4)	0.052
Mean R-VAS decrease	3.88 (±2.9)	4.44 (±3.3)	0.076
*Missing*1-month *evaluations*	*5*	*6*	

ODI: Oswestry disability index; MCID: L-VAS: visual analog scale for lumbar pain; R-VAS: visual analog scale for radicular pain.

**Table 4:** Clinical evaluation at 3 months postoperatively in the Open and UBE groups.

	Open group n = 151	UBE group n = 151	p-value
ODI (/100)	13.7 (±13.2)	15.7 (±16.4)	0.49
Mean ODI decrease	33.9 (±19.8)	32.4 (±21.3)	0.964
MCID reached	120 (90.2 %)	116 (84.1 %)	0.183
L-VAS (/10)	2.6 (±2.4)	3.0 (±2.5)	0.202
Mean L-VAS decrease	3.4 (±2.8)	3.2 (±3.4)	0.882
R-VAS (/10)	2.6 (±2.5)	2.5 (±2.6)	0.483
Mean R-VAS decrease	4.6 (±2.8)	4.7 (±3.5)	0.444
Satisfied or very satisfied	124 (89.9 %)	126 (86.3 %)	0.664
Percentage of improvement reported by patients	80.9 (±17.6)	80.5 (±20.4)	0.441
Return to work (active patients)	59 (55.1 %)	52 (50.5 %)	0.591
*Missing*3-month *evaluations*	*13*	*5*	

ODI: Oswestry disability index; MCID: minimal clinically important difference; L-VAS: visual analog scale for lumbar pain; R-VAS: visual analog scale for radicular pain.

**Table 5:** Clinical evaluation at 1 year postoperatively in the Open and UBE groups.

	Open group n = 151	UBE group n = 151	p-value
ODI (/100)	14.5 (±15.1)	13.8 (±15.3)	0.518
Mean ODI decrease	33.2 (±20.9)	33.8 (±21.7)	0.807
MCID reached	102 (85.7 %)	100 (86.2 %)	>0.999
L-VAS (/10)	3.1 (±2.7)	3.2 (±2.8)	0.313
Mean L-VAS decrease	3.0 (±2.8)	2.9 (±3.5)	0.827
R-VAS (/10)	2.9 (±2.8)	2.6 (±2.8)	0.313
Mean R-VAS decrease	4.4 (±3.2)	4.7 (±3.4)	0.499
Satisfied or very satisfied	109 (97.3 %)	110 (98.2 %)	>0.999
Percentage of improvement reported by patients	79.0 (±22.7)	81.6 (±21.1)	0.311
Return to work (active patients)	83 (90.2 %)	71 (83.5 %)	0.272
Work delay after surgery (days)	96.8 (±75.7)	91.0 (±95.7)	0.093
*Missing 1-year evaluations*	*27*	*30*	

ODI: Oswestry disability index; MCID: minimal clinically important difference; L-VAS: visual analog scale for lumbar pain; R-VAS: visual analog scale for radicular pain.

**Table 6:** Early and late complications in the Open and UBE groups.

	Open group n = 151	UBE group n = 151	p-value
Perioperative complications	5 (3.3 %)	6 (4.0 %)^a^	>0.999
Dural tear	4 (2.7 %)	4 (2.7 %)	>0.999
Including conversion	1 (0.7 %)	2 (1.3 %)	>0.999
Retroperitoneal water diffusion	0	1 (0.7 %)	>0.999
Immediate resolutive confusion	0	2 (1.3 %)	0.498
Secondary complications	9 (6.0 %)	12 (8.0 %)	0.651
Hematoma	0	3 (2.0 %)	0.248
Hernia recurrence	6 (4.0 %)	7 (4.6 %)	>0.999
Same level reoperations at 1 year	9 (5.9 %)	10 (6.6 %)	>0.999
For hematoma	0	2 (1.3 %)	0.498
For hernia recurrence	6 (4.0 %)	6 (4.0)	>0.999
Arthrodesis	3 (2.0 %)	2 (1.3 %)	>0.999

a 1 patient from the UBE group experienced 2 perioperative complications: dural tear and immediate resolutive confusion.

**Table 7:** Perioperative times and cost data for the Open and UBE groups.

	Open group n = 151	UBE group n = 151	p-value
Operative time (min)	44.0 (±15.0)	57.2 (±19.5)	<0.001*
Consumables (€)	91.2 (±90.6)	260.6 (±89.1)	<0.001*
Hospital stay (days) Ambulatory	1.2 (±0.9) 14 (9.3 %)	1.1 (±0.9) 31 (20.5 %)	0.14 0.01*

### 3.3 Patient satisfaction and professional activity (Table 4, Table 5)

At M3 and M12, most patients in UBE and Open groups were satisfied or very satisfied with their surgery, with no significant difference between groups (86.3 % vs. 89.9 % f at M3, p = 0.664, up to 98.2 % vs. 97.3 % at M12, p > 0.999). Patients in both groups described similar alleviation of their symptoms, about 80 % at both times (M3 and M12). The two groups did not differ in rate of active patients who had returned to work at M3 or M12.

### 3.4 Complications and reoperations (Table 6)

The two groups did not differ in complication rate, regardless of complication (accidental durotomy, revision for hematoma, or revision for LDH recurrence). In the immediate postoperative period, two patients in the UBE group experienced an episode of neurological confusion and agitation, which resolved rapidly; in one case, this occurred following a dural tear. No surgical site infection was observed. At 1 year after surgery, the rate of hernia recurrence did not differ between the Open and UBE groups (6 vs. 7 patients, p > 0.999) nor did the rate of patients requiring another surgery at the same level (9 vs. 10 patients, p > 0.999).

### 3.5 Operating and hospitalization times and costs (Table 7)

The mean operating time was longer for the UBE than Open group (57 vs. 44 min, p < 0.001). The cost of disposable materials was significantly higher with UBE than Open surgery (mean 261€ vs. 91€, p < 0.001), but the average hospital stay was similar (mean 1.1 vs. 1.2 days, p = 0.14). The outpatient rate was higher for the UBE than Open group (20.5 % vs. 9.3 %, p = 0.01).

## 4 Discussion

The objective of the study was to compare mid- and long-term results for LDH treatment by UBE or microdiscectomy. The results of this study demonstrate that LDH treatment with the UBE technique, even in the early stages of a surgeon’s practice, is effective, safe, and yields at least as good results as microdiscectomy.

### 4.1 Clinical outcomes

A previous randomized trial by Park et al. (2023a) compared clinical outcomes between the two techniques and found similar results except for surgical site pain, which was slightly lower in the UBE than microdiscectomy group up to 48 h postoperatively (mean difference in VAS pain score −0.98 [95 % CI -1.77 to −0.19]).

We had hypothesized that back pain would be better in the UBE than Open group. Our results do not support this. However, other studies highlight the reduced back pain with UBE as compared with conventional surgery (Kim et al., 2018; Chang et al., 2023).

Both techniques were effective in reducing pain and disability as reflected by changes in ODI, L-VAS, and R-VAS at M1, M3, and M12. Additionally, a high proportion of patients reached the MCID without any difference between groups. Previous reports documented the superiority of surgery versus conservative treatment for LDH, especially in the short term (Gugliotta et al., 2016). Our results reflect that the plateau in clinical improvement for both techniques is reached at 3 months and maintained during follow-up (Fig. 1).

The UBE and Open groups were comparable in satisfaction (86.3 % vs. 89.9 %, p = 0.664) and return-to-work rates (50.5 % vs. 55.7 %, p = 0.541). Regarding return to work, our outcomes could be criticized because our postoperative protocol does not differ between the two techniques.

### 4.2 Complications

We found no significant differences in complication or reoperation rates between the groups. This highlights the safety profile of UBE, even during the initial stages of the surgeons’ learning curve. The absence of surgical site infections in both groups further emphasizes the reliability of both techniques, even if hypothetically the permanent water outflow in endoscopy is supposed to limit the risk for infection even further.

The two groups did not differ in incidental durotomies. However, the rate of dural tears could be underestimated in the UBE group. The overall incidence of dural tears in UBE surgery is about 2.5 % (Park et al., 2023b; Wang et al., 2023). Care should be taken in endoscopy because the water environment and its pressure on the dural sac can lead to missed dural tears. However, because of the relative hermetic work space, which restricts cerebrospinal fluid leak, potential tears in the UBE group, if existing, were not symptomatic.

Lastly, two patients in the UBE group experienced immediate confusion and agitation without sequelae. We hypothesize that these complications were caused by an increase in intracranial pressure in case of a dural tear. A dural tear was identified in one of these cases; in the other, it is possible that we missed it. This type of complication has been described in the literature (Zhang et al., 2022; Heo et al., 2022). Following these observations, and in accordance with the literature (Ju and Lee, 2023), we decided to discontinue the use of the pump.

### 4.3 Surgical time, learning curve, hospitalization, and associated costs

Operative time is an important topic. At first glance, it may seem difficult to advocate a technique that provides similar outcomes but requires longer operative times. In our results, use of the Open technique resulted in a shorter mean operating time than did UBE (44 vs. 57 min, p < 0.001), which contrasts with the literature, which deems biportal endoscopy as fast as microdiscectomy and monoportal/full endoscopic surgery (Choi et al., 2019). However, given that all three surgeons were at the beginning of their learning curve in UBE during this study, a 13-min difference could be considered not substantial. An analysis comparing both techniques after the learning phase is necessary. Recent analysis (Xu et al., 2022) places the number of interventions for mastering UBE surgery at about 54 cases. Furthermore, this is the first study to our knowledge to portray results of a real-life scenario in which trained surgeons transition from one technique to another.

In terms of the cost of disposable materials, UBE surgery was more expensive in the French health system (mean 261€ vs. 91€, p < 0.001). The primary difference was from the cost of both radiofrequency probes and burr tips. This higher cost also seems to be the case in other health systems, such as China (Chang et al., 2023).

The literature suggests a near-zero infection rate with endoscopy techniques, and rates from 1 % to 2 % with Open surgery (Kang et al., 2021; Habiba et al., 2017). The cost of additional surgery, prolonged hospitalization, and antibiotic treatment can rapidly weigh in the economic balance. We also observed an increased rate of ambulatory surgeries with the UBE technique, which can lead to lower hospitalization costs; these observations were supported by a Chinese cost-effective analysis (Yang et al., 2024) and need to be confirmed by other studies worldwide.

### 4.4 Limitations

Although these results provide evidence that UBE surgery is as effective as standard microdiscectomy, there are limitations in the study. The initial population for this study was not comparable in terms of ODI and R-VAS perhaps because of surgeon selection: voluminous and extruded disk herniations that are clinically more significant are the best target at the start of learning curve in UBE surgery. Posterior propensity matching should solve this issue. Additionally, the cost-effectiveness findings reported in this study may not be generalizable to other countries, due to differences in healthcare organization and public health systems. Finally, despite a fairly large sample and long follow-up, this remains a retrospective and single-center study, which could limit the generalization of our findings.

The predominance of patients with left-sided pathology in the UBE group is also a limitation, as it may artificially reduce the complications rate. The issue of hand dominance is specific to biportal endoscopy, where visualization and working portals are separated. During the early learning curve, right-handed surgeons often prefer left-sided pathology, as it allows the endoscope to be held in the non-dominant hand while the dominant hand is used for dissection and instrumentation in the safest and least time consuming way possible. But when the learning curve is achieved, it is common to approach the disc herniation from the same side as the pathology. Three main strategies are usually used: first, the surgeon may remain on the left side of the patient and operate in a mirror-like fashion, although this can require greater manipulation of neural elements. Second, the surgeon may stand on the right side and invert hand roles, holding the endoscope in the dominant right hand and operating with the non-dominant left hand, often switching during neural handling after drilling is completed. Third, the surgeon may also stand on the right side while maintaining the endoscope in the left hand; however, this requires the portals to be placed lower, which can reduce the working angle and increase the risk of over-drilling to achieve adequate visualization. It is also worth noting that some surgeons describe contralateral approach through the canal for specific disc herniations.

## 5 Conclusions

This study shows that the UBE technique is at least as effective as conventional microdiscectomy for LDH, even when comparing clinical results from the start of the learning curve. These results suggest that surgeons can profit from training in UBE for simple cases such as lumbar discectomies without sacrificing patient safety. This training allows surgeons to transition to more complex surgeries within endoscopy for which clinical superiority might be more noticeable.

## Funding source

This study did not receive any financial support.

## Declaration of competing interest

The authors declare that they have no known competing financial interests or personal relationships that could have appeared to influence the work reported in this paper.
